# I Do It, but I Decide With Whom

**DOI:** 10.3389/fpsyg.2021.673617

**Published:** 2021-08-05

**Authors:** Cristina M. Pulido, Ana Vidu, Sandra Racionero-Plaza, Lídia Puigvert

**Affiliations:** ^1^Department of Journalism and Communication Sciences, Autonomous University of Barcelona, Cerdanyola del Valles, Spain; ^2^Department of Private Law, University of Deusto, Bilbao, Spain; ^3^Department of Sociology, University of Barcelona, Barcelona, Spain

**Keywords:** social interaction, autobiographical memory, language of desire, new alternative masculinities (NAM), dominant traditional masculinities (DTM), memory reconstruction

## Abstract

Social interactions and communication shape the desires and preferences of men and women. While it is true that some men have modified their behavior due to feminist women, the same happened with some women, who changed attraction patterns thanks to new alternative masculinities (NAM). This study examines the latter, focusing on social interactions mediated by language, as a crucial element to impact and change the desires of people. For this purpose, six autobiographical interviews were conducted with women aged 19–39 years, from two different countries and continents, paying attention to the narratives of their sexual-affective relationships. Using the communicative methodology, interactions have been analyzed from verbal communication and nonverbal communication, based on the consequences of the actions rather than intentionality. The results of this study show how dialogic communicative acts with NAMs influenced some women who first defended or justified actions of male perpetrators to later prefer to support female survivors against their perpetrators. Analysis reveals that communicative acts grounded in such language that enacted the desire of NAM for women of solidarity have shaped some memories of women of relationships with dominant traditional masculinities (DTM) and, ultimately, contributed to change their attraction and election patterns.

## Introduction

Scientific research studies in social interaction and communication have shown their potential in the acquisition and modeling of social behavior. There is an agreement in the literature studies on the fact that what makes us human is our ability to interact with other people (Mead, 1967). We are social beings, and we develop in society acquiring tastes and desires through the process of socialization. This implies that we know that human behaviors are created in society and are also transformed in society. A recent research study has even linked the influence of social interaction on the brain and memory (Hirst and Rajaram, 2014; Racionero-Plaza et al., 2020). Given that the main function of memory is about the future and not about the past (Kandel et al., 2013), it is key to analyze those communicative acts that make us remember wishes and preferences. Current advances in neuroscience show that not only human behaviors are able to be transformed but also human memory and the memories of people could be transformed as well (Williams et al., 2008).

Gender-based violence (GBV) and its effect on the health of the people, both at an economic level and social level, as well as physical level or mental health level, are a problem that affects many social domains (Ramón y Cajal, 1989). Furthermore, violence among adolescents is increasing and dating violence is of increasing concern (Puigvert et al., 2019). This study analyzes the socializing processes, the dominant coercive discourse, and the preventive socialization of GBV. Along with the positive insights regarding social change, this study focuses on the transformation of memories of violent relationships. The language of desire (López de Aguileta et al., 2020) is studied as a channel for this transformation, through communicative action with men who perpetuate attitudes of new alternative masculinities (NAM) (Flecha et al., 2013). This new line in the analysis of masculinities opens the way to changing the attractiveness patterns that the dominant coercive discourse tries to impose through violent attitudes portrayed as attractive.

## State of the Art

### Communicative Acts and the Self

The concept of “communicative acts” (Soler-Gallart, 2017) highlights “we do things” not only with words (Austin, 1962) but also with myriad symbols involved in *nonverbal* communication (tone, facial expression, and gesture, etc.), which may communicate on their own or accompanied by words. In addition, the theory of communicative acts has shed light on the importance of *context* and *effects* of communication because of their influence in the construction of a wide range of social phenomena. Certain phrases, such as “look at me,” acquire a different meaning if they are performed by a boy in a disco and addressed to a girl who he does not meet before, or they are said by an ophthalmologist who is examining the eyes of a patient in a clinic. Accordingly, those who interact with one another should be aware of context to make sure that their communicative acts do not produce undesired effects. Another central feature of the approach of communicative acts is the importance of the consequences of communication rather than the intention of speakers. In this sense, the theory of communicative acts goes beyond the philosophy of consciousness (Habermas, 1987) to include the relevance of interaction, to emphasize that what makes a difference in terms of social reality are the consequences of communication. Following this implication, people interacting with each other should care about the effects and consequences their communicative acts might produce on the other person, rather than be concerned about their intentions when communicating. The context and effects of communication are of particular analytical relevance in this study.

Interaction, as the genesis of communication, is also central to the theory of communicative acts for understanding the development of both this social phenomenon and the self. Building upon the symbolic interactionism of Mead (1967), the approach of communicative acts agrees on the fact that it is through communication with others, mediated by linguistics and socially constructed signs, that we build an image of ourselves (Mead, 1967). The Self is in constant dialog with two phases: the “I,” which is the personal and most organic reaction to the internalization of the perceptions of others of us, and the “Me,” the internalization of the views of significant others into the Self. Among the views of other people, those about expectations, beliefs, and emotions related to us are transmitted by communicative interaction and are individually internalized, thereby developing our sense of the Self. The communicative development of the self and its transformation frame part of the goals of this study.

Based on the community in which one develops and the history of social interactions someone has had, people use to internalize a particular idea of what is expected from us as humans, what is valued in our communities, how we should behave, and personal perception of our own Self (Wertsch, 1993). Linking this approach to the sexual-affective sphere, some women who have been socialized in a context where men are considered as attractive, fits into the category of dominant traditional masculinities (DTM) (Flecha et al., 2013), and it is expected, for some women, to internalize a positive view of themselves as attractive too. This may happen especially considering they might have had some kind of sexual-affective experience with DTM, and their peers reinforce this idea for them (Castro and Mara, 2014). Nonetheless, research studies in the area of preventive socialization of GBV have examined such phenomenon and described it as a “mirage of upward mobility” (Rosell et al., 2014). This concept describes the misunderstanding reality experienced by a girl or woman who believes she increases her attractiveness by having sexual-affective relationships with DTM and when she behaves so to draw their attention, while the contrary happens and she uses to become less attractive for DTM and for other men. Unless that woman does not experience other, very different, communicative interactions, she may tend to repeat that behavior with other men, whether they are DTM, oppressed traditional masculinities (OTM), or NAM (Gómez, 2015; Joanpere and Morlà, 2019). The research works reported in this study explores the tendency of this behavior in some women when interacting with men considered NAM and, more specifically, the response in terms of communicative acts that such behavior evokes in NAM. Additionally, our study examines the potential of the communicative acts of NAM to reconstruct the memories of these women of their relationships with DTM, voiding them of attractiveness and increasing the attraction of women to NAM.

### The “Social Turn” in Memory Research Studies: The Role of Communicative Interaction in Memory Development

Research studies on the *social turn in memory* (Hirst and Rajaram, 2014) emphasize the constructive nature of autobiographical memory, that is, the type of long-term memory of the events of the own life of an individual (Conway and Holmes, 2004). In particular, the approach of this research study stresses the influence of social interaction in recreating episodic memories, which are memories that we employ to remember past events we have experienced (Loftus, 2013). This perspective is inseparable from the understanding of mental life as “acts of meaning,” which are collaborative and communicative in nature (Bruner, 1990).

Studies in this field have noted the importance of group dynamics and types of interactions in shaping “collective memory” (Halbwachs, 1992) and the power of social interaction to implant a “memory” into another person (Loftus, 2013). More precisely, in terms of communicative acts, it is noted that people used to remember things in a collaborative way and through communicative action. All these research works show that social interaction is not only central to encoding information but also to the recalling and rebuilding of autobiographical memories (Marian and Neisser, 2000; Hedrick et al., 2009). Along this line, the previous studies have indicated the importance of context in enhancing memory retrieval, including the linguistic context. In particular, it has been shown that information acquired in a certain linguistic atmosphere is likely to become more accessible when recall takes place in that same ambiance (Marian and Neisser, 2000).

Our study included this social perspective on memory, understanding that attractiveness has a social origin (Gómez, 2015), so the attraction to certain types of men is influenced by the way in which individuals remember sexual-affective episodes, i.e., with desire or disgust. In this regard, we explored the extent to which men, with characteristics of NAM, use a language of desire in their communicative interactions with some women might be able to promote the following: (a) the retrieval of memories of women of sexual-affective experiences with DTM and (b) the reconstruction of women of some of those autobiographical memories regarding their associated emotion and self-perception.

In addition, a research study has indicated that autobiographical memory is a constituent part of the self-memory system (Williams et al., 2008; Kandel et al., 2013). Memories become the knowledge base providing a sense of Self and coherence in our life; at the same time, they are key to personal interpretations of new experiences and to decision-making (Klein et al., 2010). Thus, if occurring that some women change to some extent their perception and memory of sexual-affective relationships with DTM through communicative interaction with NAM, this might well foster other transformations in attraction and election patterns supporting intimate relationships free of violence. Because of such potential effects, the research study reported here is aligned with studies on positive elements that support constructive human development (Vázquez et al., 2009); in our case, language and interaction are conceptualized as useful tools that can support better sexual-affective relationships, thus letting people act in the world in order to transform it (Coulthard, 2011).

### Language of Ethics to Combat the Coercive Dominant Discourse

The coercive dominant discourse has been defined as contributing to promoting the attraction toward violence (Racionero-Plaza et al., 2020), through which people with violent attitudes tend to be socially presented as attractive models, while people whose behaviors are not violent are usually presented as less attractive (Gómez, 2015). A research study has also shown that violent profiles, or boys belonging to the so-called hegemonic model of masculinity (Connell, 2012), tend to be the ones most preferred by girls for sporadic relationships, while boys with a nonviolent profile are usually chosen for stable relationships (Puigvert et al., 2019).

Communicative acts are a key element in the analysis of the influence of the dominant coercive discourse. In this sense, the concept of “mirage of upward mobility” has also been analyzed (Rosell et al., 2014). It is defined as the erroneous perception that some girls have developed when they believe that, if they have a sexual relationship with a boy with violent attitudes, this fact increases her attractiveness or her status, while research studies have shown that the opposite actually happens, that is, her status decreases.

An interesting aspect of dialog is framed under what is described as “language of desire” or “language of ethics” (López de Aguileta et al., 2020). The *language of ethics* refers to attitudes defined as *good*, *convenient*, or that “*should be done*.” Otherwise, the *language of desire* used to be performed when linking attractiveness to violent behavior. In the cases analyzed in this study, the language of ethics would be implied to refer to some boys as bored or unattractive, especially those with good behaviors, while the language of desire would be used to mention some behavior as cool and desirables, especially when performed by those boys that places as “for sporadic relationships” (Gómez, 2015).

## Materials and Methods

The research works discussed in this study employed the communicative methodology of research studies (Redondo-Sama et al., 2020; Gómez-González, 2021), which has been previously used in research studies on communicative acts that achieved important scientific and social impact (Soler-Gallart, 2017; Rios-Gonzalez et al., 2018). A central feature of the communicative methodology is that it is not limited to describing social reality but also provides mechanisms for solving social problems. Such transformative scientific knowledge results from another key feature of the communicative methodology, the constant dialog between researchers and participants in the research studies. Researchers share scientific knowledge on the topic being researched, which is later contrasted with everyday knowledge from the research participants. It is in that dialog between the two types of knowledge, scientific and experiential, that it is possible not only to better understand social problems but also to contribute to their transformation (Denzin and Lincoln(eds), 2011; Gómez-González, 2021). In our study, the dialog between researchers and participants involved ongoing conversation in which we shared primary knowledge from the study of communicative acts, gender violence, masculinities, and memory, and the participant women provided their interpretations about the topic of communicative acts, desires, and memories of men.

### Sample

Participants were selected after a series of autobiographical interviews focused on their history of sexual and affective relationships. These interviews included questions and examples that helped the potential participants to identify whether such history included relationships or interactions with men who exhibited some characteristics in line with what the study has conceptualized as DTM and NAM. On the one side, the model of DTM is embodied by those men who represent the values of the patriarchal society, sometimes related to violent behaviors, and use to envision themselves as “experts” on sexual issues. On the other side, the model of NAM constitutes a utopian model, featured by the language of desire and embraced by men who are equalitarian, against violent attitudes, and are considered sexually attractive at the same time (Puigvert et al., 2019). Six women constituted our sample. They were from two different countries, Spain (*n* = 2) and Chile (*n* = 4). Their age ranged between 19 and 39 years old, and most of them had completed a degree in higher education.

### Data Collection

In-depth interviews with a communicative orientation were conducted individually with each woman. Before the interviews, the researcher explained the role and made themselves clear about the different models (as “ideal types”) of masculinity found in the scientific literature study, emphasizing their characteristics regarding their behaviors toward women (Flecha et al., 2013). Following the premise of communicative methodology, the authors shared the objectives of the study being conducted. Participants had a chance to ask questions if they wanted. In addition, researchers employed examples to clarify the aim of the questions. When responding to the specific questions during the interview, the participants themselves decided which personal stories to share and which elements to select from their memories of past sexual-affective relationships.

The in-depth interviews lasted approximately 90 min each. The participants signed a consent form, meaning a written document consenting to the use of the data in a rigorous scientific manner while remaining entirely anonymous. All interviews were conducted in Spanish, and they were later translated into English by the same researchers who carried out the interviews.

### Data Analysis

The verbal data collected by the authors were transcribed and analyzed according to the theory of communicative acts, and the main questions of the study were as follows: How do men, who (according to the participants) might correspond to some of the characteristics of the NAM category, exert influence with their “language of desire” over women who might have defended DTM? More concretely, how do the communicative acts performed by those men influence these perceptions of women regarding previous sexual-affective relationships with DTM? Additionally, how does such influence, if any, affect the memories of the woman with whom the NAM interacts in terms of how each of them remembers potential relationships with DTM?

To respond to the aforementioned questions, the authors analyzed the content and the consequences of communication in terms of the impact on the self-perceptions, memories, and behaviors of women.

This study was submitted for evaluation by the CREA Ethical Committee and approved under the reference code: 20210225.

## Results

Once the interviews with the six women were carefully analyzed, two main findings emerged as particularly relevant. On the one hand, the authors could confirm that the communicative acts performed by those men, who the female participants perceived as NAM, contributed to the questioning and dismantling of the sexual-affective perceptions and attitudes of participant women about the experiences of women in the past with DTM. On the other hand, the data showed that the communicative acts performed by NAM, grounded in the language of desire, could drive the recall of memories of women of past sexual-affective relationships, supporting a review of those memories regarding their associated emotions, and promoting a shift from attraction to rejection of DTM. This trend of change seemed to generate a reflection among these women regarding changes in their behavior in relation to DTM, to NAM, and to other women, thereby increasing feminine solidarity.

### “There Are Many Who Would Never Like to Meet Up With Him”: Communicative Acts of NAM Based on the Language of Desire Dismantling the Perceptions of Women

Following the theory of communicative acts, we focused our analysis on the *contexts* and *effects* of communicative acts performed by those men who the participants perceived as or closer to NAM. Findings indicated that interactions with men, who the participant women defined as embodying NAM characteristics, developed in a *context* featured by the language of desire. In addition, authors could identify that the central feature of those communicative acts was the *effect* they produced in women, impacting the beliefs of women about the previous sexual-affective behavior in relation to those men they perceived as DTM and supporting a change in how they visualized themselves in such past situations.

Monica shared a story in which she was engaged in a relationship with a guy whose features she presented as a DTM. She broke up with him, but they continued to call each other and meet occasionally. One day, Monica wondered whether she was getting together with him after he had called her. Monica wanted to meet him, but she was having doubts because she knew that he was not the “right” guy. She decided to talk about it with a male friend, Paul, whom she defined as NAM:

I remember a guy with whom I was engaged in a relationship. After we broke up, he continued to call me occasionally. One day he called me to ask for a meeting with him, and I was wondering whether to do it or not. So, telling this to a friend, I made a comment like “**any girl would want to go out with him**.” Then, my friend said, **“That’s not true, there are many women who would never like to meet up with him.” He said it in a way that deeply touched me**. It was not only the message that other girls would never be with that other guy but also that those girls who desire to meet up with him are crappy. **The real meaning of his words was not that “you say ‘yes’ and other women would say ‘no’, but, ‘you are the crappy one that would be able to meet up with him, because I know girls who would never meet up with him.**” And, for sure, the way of saying it, because the sentence itself was nothing special (…) But the sentence: “all girls would like to meet up with him” had something behind, the idea that “I am cool because I want to be with him.” And his response: “not all women would like to meet up with him” had a different message behind: “**not all are so crappy like you.”**

This story shows that Monica desired to meet up with the man she defined as a DTM. To express her own desire, she used the argument that all women would desire to do the same. She responded very naturally with that idea in her interaction with her friend. Immediately, he replied with an answer that made two things clear to her: that the desire of Monica was not representative of the desire of other women and, also, that he did not like women who desired to meet up with men like that guy.

We do not know the intentions the male friend had behind his words, but his communicative act made Monica think that she appeared ridiculous in the eyes of Paul. That effect was central in her revision of her self-perception with a different insight: “*my statement: ‘all girls would like to meet up with him’ had something behind, the following idea: “I am cool because I want to be with that boy.” And his response: ‘not all women would like to meet up with him’ had a different message behind, as follows: ‘not all are so crappy like you’.*” Instead of making her see herself as “cool” for having the opportunity to meet up with that guy, he made her think that maybe she was “crappy.” Additionally, the way the interviewee shared the episode, ending with “*And his response: ‘not all women would like to meet up with him’ had a different message behind—‘not all are so crappy like you”’* may indicate that she did feel crappy at that moment of the interaction, and as she told us, she worried about looking crappy in the eyes of her friend, who purported to know in person, many women who would never go out with a guy like that: *“I know girls who would never meet up with him.”*

The interviews revealed other situations that showed how some responses of men caused some women to question their perceptions of their sexual-affective behaviors, which they may have learned in the previous experiences with DTM. In this regard, Monica also described a situation in which she was dating a boy whom she defined as close to NAM in the interview. She liked him and wanted to start a romantic relationship with him. However, according to her, he did not show much enthusiasm, despite Monica manifesting she was interested in him when they got together. She thought that maybe he was not aware that she was available, and more broadly, she thought he “did not get the message.” Therefore, Monica decided to be explicit and tell him that she liked him, which generated a response from the man that shocked her:

I remember one boy that was NAM; I went out with him and we were meeting and so on… but there was no feedback on his side… And I thought that maybe he just did not realize that I liked him, **he was just a little “short”** (meaning he wasn’t very smart) **and did not get that he could go out to me; I mean that I was available and he could flirt with me**. One day we were talking about people who we liked and so on, and I said: “**I like you too, eh,”** and he said: “**I’ve already noticed.”** And I was like, **“So what? Do you like me too?”** He said: “**Well, a little, but that does not mean we are going to have sex. I know I can, but I’m deciding if I want to**.”

The reflections of Monica show the impact of her socialization with DTM on how she interpreted the situation with this other man, whom she defines as NAM in the interview, and how she perceives and talks about him. Her flirting strategies, as learned in prior relationships with DTM, do not work with the NAM. Instead of considering the possibility that she was doing something wrong, she believes that the problem was the man, who *“was just a little ‘short’ and did not get that he could start a relationship with me.”* Monica perceived the man as out of place in the flirting game, as if he was poorly experienced. However, the words of the man showed a radically opposite situation: *“I know I can, but I’m deciding if I want to.”* With just a few words using the language of desire, he changed the mind of Monica, as she shared:

You can imagine, someone who you know 100% to be nice, he is NAM, is telling you that! That dismantles you, and not because a similar situation had never happened to me; actually, it already happened, that guys had “gone away” from me. However, this guy is someone super cool, nice, and it is clear that he likes me, but it is just… he does not like how I’m behaving. It’s as though “**with this attitude… I do not want to be with you.**” Then, I thought, what do I have to do to make him like me? **Then, I realized that what I was used to doing when flirting with boys was not working anymore.**

With the response of this man, Monica realized not only that she was wrong in her analysis, as he confirmed that he had noticed that she liked him, but also, and more importantly, she realized that it was her who was out of place in the game of flirting. As she expressed: “*I realized that what I was used to doing when flirting with boys was not working anymore.*” So, Monica understood that with the man whom she defined as NAM she used the flirting strategies she had used before with DTM.

I was misplaced because I was not used to that type of answer. Someone nice who tells you, “**Yes, I like you, but I cannot see the point of your behavior.**” Therefore, I felt like, “What do I have to do in order to get a yes from him?” I mean, what do I have to do to get him to have more and more desire and interest [for me]? That was a key moment of transformation for me.

The clarity and confidence in the words of the man Monica described as NAM made her question her behavior while increasing her desire for him. This produced a change in the behavior of Monica from that moment on:

Then, I decided to do nothing, just to be natural. That was when we got on, and I was like: “Now?” For me, it did not fit at that moment. I thought, “Why now and not before?”, but it was then when it happened.

The *results* of the communicative act performed by that man made the woman change her behavior, to “just be natural,” and not engage in any strategies. Monica stopped pressing him, and thus, he liked her more and decided to move forward.

Another participant, Luisa, described a situation in which she behaved antagonistically toward a man with whom she had a relationship and had defined as NAM in the interview. She shared that this man was helping her to finish some paperwork she had to do before an imminent deadline. The man had decided to stay up late into the night to help her. At one point, Luisa reacted in a very negative way, complaining about what he was doing. Right after, the communicative acts performed by the man, both verbal and nonverbal, were very convincing, strong, and clear:

He was losing sleep for me. He decided to be there to help me to finish things, for me to have everything ready and well done. And there was a moment in which I talked badly to him: “You are doing this wrong! This thing does not go there, it goes here! You do not understand!” When that was a thing for me, and he was helping me and being very much in solidarity with me… And he said: “**OK, so, I’ll leave you. I’ll go to sleep. You take care of it by yourself.”** And he left the room and left me there alone. (…) And he did not help me anymore, but not because he was not in solidarity with me. That made me think that I could not treat people that way; that people are not going to tolerate me in that way marked by a lack of solidarity. I have not done that anymore, as I saw that the next time he would not go to sleep but he would leave home.

She continued her reflection sharing the importance of the language of desire expressed not only in the words of the man but also in his gestures, facial expressions, looks, and tone, etc. This can be seen in the following explanation of Luisa of her own communicative acts when recriminating her boyfriend and when she reiterates that what made her conscious of the seriousness of what happened was, above all, the looks and facial expressions of her boyfriend and the fact that he left her alone in the room:

In that situation, I was telling him all those things **and I was not looking at him or anything, and using a very disgusted tone of voice**. And **he looked at me as if he was telling me: “I have no desire for you.” His was an insight that expressed clearly that he was making no effort to tell me “bye” but, on the contrary, it was like: “Sorry, girl? I don’t like this at all**.” So, it was not only what he said… I saw he was not seeing me as attractive, that he was seeing me as a rude person, a woman he does not want to be with. So, what he said impacted me, but much more so was his way of looking at me, which told me “this is not the girl I want to be with.” It **made me think a lot… not only because of what he said but also because he left the room**. So what he said was accompanied by action; he was absolutely coherent because he sincerely felt that rejection. He did not make any effort; he did so because he really did not like me that way.

Here is evidenced that the power of the communicative acts of NAM grounded in the “language of desire” to impact perceptions and that the behavior is not limited to words: *So what he said impacted me, but much more so was his way of looking at me, which told me “this is not the girl I want to be with*.” The effects of the communicative interaction, translated in the fact that the man left the room leaving her alone, were acknowledged by Luisa as central in making her aware of how disgusting was her lack of solidary behavior toward him: *It made me think a lot… not only because of what he said but also because he left the room. So what he said was accompanied by action; he was absolutely coherent because he sincerely felt that rejection.*

### “(It) Made Me Change the Perception of That Entire Memory”: The Power of the Language of Desire of NAM to Trigger Memory Revision and Reconstruction

The analysis of the in-depth interviews sheds light upon another central finding: The language of desire of the communicative acts performed by the men who the interviewees perceived as NAM made these women question their perception of the relationships with DTM men and their behavior in those relationships. Such questioning meant a turning point in terms of the beginning of the revision of memories of those past sexual-affective relationships, fostering their reconstruction by initiating a process of emptying them of attractiveness.

Monica shared a story in which she was engaged in a relationship with a guy from a village where she used to spend the summer when she was an adolescent. She defined this man as DTM. They were together one summer, while he had a girlfriend and Monica had a boyfriend. After the summer, she returned home and continued her life and relationship with her boyfriend. Monica had always considered that boy from the village to be a friend. Some years later, while she was initiating a different relationship with a different man who she labeled as NAM, the boy from the summer village phoned to invite her to his wedding. He was getting married to the same woman he had betrayed that summer when he and Monica were together. She could not understand why he was inviting her to his wedding with that girl, yet somehow she was happy to be invited by a man she still considered a friend. She decided to share this circumstance with her current boyfriend. His communicative acts after hearing Monica tell the story were strong and definitive:

I explained it to my boyfriend. He did not know the whole story, and I told him at that time. Then, what surprised him the most was not the fact that I was with that guy that summer, but **how I described the story: I was very happy, smiling, like “Ha, ha… That’s a friend calling me… Ha ha ha! And I am not going to go to his wedding in which he marries that girl.”** Then, he [the boyfriend] stood up with such a face that I freaked, and I thought: “Do not be shocked! It is not so shocking. Moreover, I am not going to attend the wedding.” After that day, we met two more times, and he was a little weird. Then, one day, **he said that he did not know why, but he was no longer in love** (…) So, I told this story to Luis, a NAM friend who clearly stated that, actually, I was the one who broke up the relationship. And I was like: “Why? It was just received a call from a colleague who invited me to his wedding.” My friend told me that **it was a stupid boy calling me 10 years later to invite me to the wedding with this girl who he had cheated on**; **and that he did so because of the morbidity attached to the infidelity**. When Luis made me see that, it changed my image of what happened.

This quotation shows that it was through the communicative acts with her boyfriend and her friend how Monica revisited and reconstructed the memories of that summer relationship. The communicative act of the boyfriend [“*He said that he did not know why, but he was no longer in love*”] and the consequences of it [ending the relationship], together with the *sincerity* of the communicative act of her friend about the analysis of what happened [“*you were the one who broke up the relationship”*], made Monica question her perception of the sexual-affective relationship with that man from the village and of the entire episode of sharing that story with her boyfriend.

Monica moved from thinking that her boyfriend was excessive in his reaction to acknowledging that her feelings about the other story caused the end of the relationship [“*Then, I was well aware of that as the reason that had actually broken the relationship”*]. Through the communicative acts grounded in the language of desire performed by the two men, Monica came to realize that her story from that summer was neither nice nor cool. Because she had been remembering that guy as a friend and had given no importance to the betrayal, she talked enthusiastically about the phone call with her boyfriend. He noticed mainly her nonverbal expressions: She was happy and she spoke with amusement and laughter, showing that she was pleased and satisfied to be invited to that wedding: *How I described the story: I was very happy, smiling, like “Ha, ha… That’s a friend calling me… Ha ha ha! And I am not going to attend his wedding, in which he marries that girl.* What mattered for the boyfriend of Monica was not that she had decided not to attend, which is what she shared verbally, but rather her lack of concern in receiving the invitation many years later, which was manifested in her nonverbal communicative acts. Therefore, he saw Monica as a doubtful partner, and, deciding to end the relationship, he showed that he did not like women who did not react more negatively to such situations.

Monica narrated how this fact changed her memory. She began to analyze the relationship with the man from the village whom she labeled as DTM and could find no evidence of friendship on it. Therefore, she asked herself why she considered that boy to be a friend. Additionally, she began to situate betrayal as the central piece of the story and the relevance of such fact had for her relationship at that moment.

**I was well aware of the reason that had actually broken the relationship**. He was a guy [the boyfriend] who was not willing to accept that. Somebody’s past can be dark, and that’s hard to accept, but you may try to say that “now” for X time you are different and that such thing would not happen again. However, when you see this stuff as something that just happened in the past but you have not reflected well on it, then a NAM will not accept it. (…) **For him, it is like, “this girl can go out with anyone at any time.”**

Because of the “language of desire” involved in the communicative acts performed by both her former boyfriend (*he said that he did not know why, but he was no longer in love*) and her friend Luis (*my friend told me that it was a stupid boy calling me 10 years later to invite me to the wedding with this girl who he had cheated on; and that he did so because of the morbidity attached to the infidelity***),** Monica reconstructed her memories of that summer relationship, that is, she and that boy were never friends, and they were cheating on their partners, which was the cornerstone of the story instead of small detail. Thus, the memory of a “crazy summer adventure” became what it was, a typical betrayal.

The review of memories of past sexual-affective relationships through communicative acts performed by men, who the participant women labeled as NAM, was also evidenced in a story shared by another woman, Melissa. She explained that she was in a relationship with a man that she defined as a DTM. This man used to judge people, including women, based on their appearance. Melissa learned to do the same and criticized people along those lines: *When I was with the DTM, he made me see people in very superficial ways.* Later, when she started a relationship with a man that she defined as NAM, he made her realize her attitude has no sense and how shabby it was. Melissa highlighted a communicative act performed by her later boyfriend in a party, which made her see herself as “ridiculous” in her behavior and comments and fostered her change:

I felt ridiculous when I started to change my way of “seeing” other people; the new way of looking at people that my boyfriend taught to me. **That changed me when he said to me: “stop that attitude with people and be more open.”**

Language of desire is expressed here in the clarity and confidence of the sentence of the man and in the use of expressions such as “be more open-minded” as, generally, it is not desirable to be labeled as a “closed person.” In addition, that sentence acquires stronger meaning as said in the context of a social event, like a party. This led Melissa to revisit her memories of the previous relationship, to see herself as ridiculous in that past behavior, and, importantly, be aware that her current boyfriend did not like her with such attitude: *He realized my attitudes when I got to know new people and that was one of the things that he did not like about me at the beginning.* Afterward, Melissa decided to act differently in present and future sexual-affective relationships:

When I was with that man [she refers to the one she referred to as DTM in the interview], I argued that such a relationship was normal, but then… a NAM telling me more and more things… made me change the perception of that entire memory (…). And that relationship came to represent what I would now not look for in a sexual-affective relationship.

The data analyzed also indicated that the communicative acts performed by men who the interviewed women saw as close to the NAM “ideal type” also fostered some of the women to be more critical of their past behavior toward other women. Luisa, for example, shared that she changed her interpretation and memories about past relationships of betrayal in which she had been involved thanks to the communicative acts performed by men whom she perceived as NAM. She shared two episodes that shed light on the power of communicative acts grounded in the language of desire on women to reject betrayal and have more solidarity with other women who are victims of the scorn of other men whom the participant women saw as responding to the DTM type:

This happened one time that we were on a train. There was a man. We were talking about various issues. He took out a list of contacts that he had, and told me: “**I would never have on my contact list a person who has betrayed someone.”** And then I said: “But, those are only the ones who have partners who betray.” And he said, “No. No. Those who betray are all the people who participate in the betrayal, whether those people be the one who betrays and has the partner or the third person involved.” I remember so well that I thought “WOW. I have participated in betrayal.” **It was at that moment that I came to realize that I had been a part of betrayal, that I had betrayed**. This man was super clear in his mind that he rejected having men or women who participate in betrayal as friends, and this guy was very very valued in the group. I remember very well that I thought, “Ugh… I do want to be with him, I do want to be her friend.” That made me think that betrayal is always betrayal, regardless of your role in it and that there are people who decide not to have friends who betray others. (…) The fact that a man who was very valued by many men and women was telling me that… that affected me a lot, and this happened because of course, I wanted to be part of his group of friends. **I did not want to be the crappy one doing those things.**

Along with this narrative, it is of particular relevance how the friend, a very cool man in the group, performed a very sincere communicative act to convey the idea of rejecting as friends people who betray. This, together with his clarification of who the people are who betray, all those who participate in it, helped Luisa to recall past stories of personal engagement in betrayal and to see herself in a different way, no longer as more attractive than the betrayed woman but as “crappy.” Importantly, Luisa came to realize that, regardless of having a boyfriend, by participating in betrayal, she also despised other women. Other stories she shared evidenced her change in attitude in this regard, becoming a supporter of those women.

## Discussion and Conclusion

Research studies on the preventive socialization of GBV (Gómez, 2015) have examined the ability of the language of desire to promote change in attraction patterns, moving from attraction to DTM to attraction to NAM. The research works reported in this study adds to that line of research studies by exploring whether and how such language, when used by men perceived as NAM, has the ability to inspire change in the perception and memories of some women of sexual-affective relationships with DTM men, contributing to the transformation of their attraction patterns, while contributing to overcome the coercive dominant discourse that associates attraction with violent behaviors.

The analysis of data from the interviews, analyzed through the lens of the theory of communicative acts (Searle and Soler, 2004), informed us that these acts, when performed by NAM and grounded in the language of desire, tend to dismantle sexual-affective attitudes and self-perceptions of some women acquired in relationships with DTM, and somehow assumed, including flirting strategies, discord with other women, and betrayal. That first moment of “dismantling” the previous images and ideas was fundamental, as it made women think for the first time that their behavior related to relationships with DTM was inappropriate, ridiculous, and totally undesirable to other men who might respond to the characteristics of NAM.

In addition, the language of desire employed by men identified by the interviewed women as NAM encouraged the participant women to revisit some of their past sexual-affective relationships with men they described as belonging to DTM features. They came to perceive those relationships in a new light, voiding them of attractiveness. Interpreting this result from a symbolic interactionism point of view, social interaction with NAM, grounded in the language of desire, may trigger the transformation of the Me of women (Mead, 1967), supporting the development of a new sexual-affective Self. This transformation implies a process in which what was previously perceived as “cool” behavior, relationships, and masculinity is now understood as “crappy.” For the particular case of the sexual-affective Self, the dimension of “desire” in communication seems to be essential in supporting this transformation of the Me. This result aligns with other studies in conversational analysis (Nyroos and Sandlund, 2014), which have shown how the same question may beget many different actions. In this study, depending on the type of language used, i.e., language imbued with desire or not, communication acquires different meanings and produces different effects on women in terms of revising perceptions and memories of past-sexual affective relationships with DTM men.

Additionally, the dimension of *effects* of communication, a distinctive feature of the theory of communicative acts (Searle and Soler, 2004), emerged as a central factor in the research works presented in this study. According to the interviewed women, consistency between the words, gestures, and actions (effects) of NAM strongly induced these women to question their sexual-affective attitudes and past relationships and promoted change, as supported by many research studies showing how communicative daily-life stories transform the analysis of women around the reasons and effects of their own cases of GBV (Marian and Neisser, 2000; Puigvert et al., 2019).

Importantly, the evidence found suggests that the communicative acts of NAM, being *convincing, sincere*, and grounded in the *language of desire*, triggered in the participant women a process of recall and revision of potential episodes of sexual-affective relationships with DTM men. In the cases studied here, such memory revision helped the women to better understand their sexual-affective biographies and question their interpretation of certain relationships with DTM, starting to perceive those as unattractive and ridiculous, while approaching to identify relationships with egalitarian men as attractive and cool. This finding adds to research studies on the social turn in memory (Hirst and Rajaram, 2014). In particular, studies on memory and language have found that information acquired in a certain linguistic ambiance is likely to become more accessible when recall takes place in that same ambiance (Marian and Neisser, 2000). Given the evidence collected in our study, we suggest that the recall and transformation of autobiographical memories about sexual-affective relationships are more likely fostered using the “language of desire” in communicative acts. The practice of the language of desire in communicative acts performed by NAM supports similar linguistic ambiance between the time the information about the experience was encoded and the recall of that same experience. This might indicate that because desire and emotion are crucial components of *episodic memory* (Conway and Holmes, 2004), it is through desire that memories of sexual-affective relationships could be potentially changed. This is an insightful finding that should be further explored with a greater and more diverse sample of women and through complementary perspectives, such as those from cognitive psychology and neuroscience.

Finally, all findings reported are central to a transformative perspective of communication and social reality. Our results shed light on which types of social interactions and communication enhance sexual-affective relationships that are free of violence and coercion. The communicative acts explored in this study tend to enhance the creation of a desire for egalitarian men and the rejection of individuals with violent and non-egalitarian values (Rios-Gonzalez et al., 2018). Given that change in emotion implies a certain change in cognitive schemata, the effects of the communicative acts of NAM reported here might aid women in potentially making better choices throughout their sexual-affective trajectories. Longitudinal studies could more deeply examine this question. Additionally, the change in the interpretation and memories of past sexual-affective experiences with DTM seems to encourage solidarity among women; some of our interviewees who had first defended DTM turned to defend women who were victims of those men. In all of these processes, men close to the definition of NAM acquire an important role in supporting the changes in women, a finding that adds more evidence to the relevance of masculinities to social change (Connell, 2012).

## Data Availability Statement

The original contributions presented in the study are included in the article/supplementary material, further inquiries can be directed to the corresponding author/s.

## Ethics Statement

The current research was submitted for evaluation by the CREA Ethical Committee and approved under the reference code: 20210225. The patients/participants provided their written informed consent to participate in this study.

## Author Contributions

SR-P and AV conducted the research and investigation process. LP contributed to the conceptualization of the study in relation to the New Masculinities line of research. CP participated in the data collection. SR-P performed the data analysis, especially focusing on the memory analysis. CP, LP, and AV contributed to the formal analyses and discussion of the data. SR-P, CP, LP, and AV collaborated in writing the manuscript, revised its content, and approved the final version submitted. All authors contributed to the article and approved the submitted version.

## Conflict of Interest

The authors declare that the research was conducted in the absence of any commercial or financial relationships that could be construed as a potential conflict of interest.

## Publisher’s Note

All claims expressed in this article are solely those of the authors and do not necessarily represent those of their affiliated organizations, or those of the publisher, the editors and the reviewers. Any product that may be evaluated in this article, or claim that may be made by its manufacturer, is not guaranteed or endorsed by the publisher.

## References

[B1] Austin J. L. (1962). *How to Do Things with Words.* Oxford: Oxford University Press.

[B2] Bruner J. (1990). *Acts of Meaning.* Cambridge, MA: Harvard University Press.

[B3] Castro M. Mara L. C. (2014). The social nature of attractiveness: how to shift attraction from the dominant traditional to alternative masculinities. *Int. Multidiscip. J. Soc. Sci.* 3 182–206. 10.4471/rimcis.2014.36

[B4] Connell R. W. (2012). Masculinity research and global change. *Masc. Soc. Change* 1 4–18. 10.4471/MCS.2012.01

[B5] Conway M. A. Holmes A. (2004). Psychosocial stages and the accessibility autobiographical memories across the life cycle. *J. Pers.* 72 461–480. 10.1111/j.0022-3506.2004.00269.x 15102035

[B6] Coulthard M. (2011). Making a difference: critical linguistic analysis in a legal context. *Pragmat. Soc.* 2 171–186. 10.1075/ps.2.2.03cou 33486653

[B7] Denzin N. K. Lincoln Y. (eds) (2011). *The Sage Handbook of Qualitative Research.* Thousand Oaks: Sage Publications.

[B8] Flecha R. Puigvert L. Rios O. (2013). The new alternative masculinities and the overcoming of gender violence. *Int. Multidiscip. J. Soc. Sci.* 2 88–113. 10.17583/rimcis.2013.612

[B9] Gómez J. (2015). *Radical Love. A Revolution for the 21st Century.* New York, NY: Peter Lang Publishing.

[B10] Gómez-González A. (2021). Science with and for society through qualitative inquiry. *Qual. Inq.* 27 10–16. 10.1177/1077800419863006

[B11] Habermas J. (1987). *Theory of Communicative Action, Volume Two: Lifeworld and System: A Critique of Functionalist Reason.* Boston: Beacon Press.

[B12] Halbwachs M. (1992). *On Collective Memory.* Chicago: University of Chicago Press. 10.7208/chicago/9780226774497.001.0001

[B13] Hedrick A. M. Catherine A. H. Ornstein P. A. (2009). Elaborative talk during and after an event: conversational style influences children’s memory reports. *J. Cogn. Dev.* 10 188–209. 10.1080/15248370903155841 25419186 PMC4238227

[B14] Hirst W. Rajaram S. (2014). Toward a social turn in memory: an introduction to a special issue on social memory. *J. Appl. Res. Memory Cogn.* 3 239–243. 10.1016/j.jarmac.2014.10.001

[B15] Joanpere M. Morlà T. (2019). Nuevas Masculinidades Alternativas, la lucha con y por el feminismo en el contexto universitario. *Masc. Soc. Change* 8 42–63. 10.17583/mcs.2019.3936

[B16] Kandel E. R. James H. S. Jessell T. M. (2013). *Principles of Neural Science.* Norwalk: Appleton & Lange.

[B17] Klein S. B. Robertson T. E. Delton A. W. (2010). Facing the future: memory as an evolved system for planning future acts. *Memory Cogn.* 38 13–22. 10.3758/MC.38.1.13 19966234 PMC3553218

[B18] Loftus E. F. (2013). 25 Years of eyewitness science…finally pays off. *Perspect. Psychol. Sci.* 8 556–557. 10.1177/1745691613500995 26173213

[B19] López de Aguileta G. Torras-Gómez E. García-Carrión R. Flecha R. (2020). The emergence of the language of desire toward nonviolent relationships during the dialogic literary gatherings. *Lang. Educ.* 34 583–598. 10.1080/09500782.2020.1801715

[B20] Marian V. Neisser U. (2000). Language-dependent recall of autobiographical memories. *J. Exp. Psychol.* 19 361–368. 10.1037//0096-3445.129.3.361 11006905

[B21] Mead G. H. (1967). *Mind, Self, and Society from the Standpoint of a Social Behaviorist.* Chicago, IL: University of Chicago Press. 10.7208/chicago/9780226516608.001.0001

[B22] Nyroos L. Sandlund E. (2014). From paper to practice: asking and responding to a standardized question item in performance appraisal interviews. *Pragmat. Soc.* 5 165–190. 10.1075/ps.5.2.01nyr 33486653

[B23] Puigvert L. Gelsthorpe L. Soler-Gallart M. Flecha R. (2019). Girls’ perceptions of boys with violent attitudes and behaviours, and of sexual attraction. *Palgrave Commun.* 5:56. 10.1057/s41599-019-0262

[B24] Racionero-Plaza S. Ugalde L. Merodio G. Gutiérrez N. (2020). ‘Architects of their own brain’. social impact of an intervention study for the prevention of gender-based violence in adolescence. *Front. Psychol.* 10:3070. 10.3389/fpsyg.2019.03070 32116875 PMC7016211

[B25] Ramón y Cajal S. (1989). *Recollections of My Life.* London: MIT Press. 10.7551/mitpress/5817.001.0001

[B26] Redondo-Sama G. Díez-Palomar J. Campdepadrós R. Morlà-Folch T. (2020). Communicative methodology. contributions to social impact assessment in psychological research. *Front. Psychol.* 11:286. 10.3389/fpsyg.2020.00286 32194474 PMC7063089

[B27] Rios-Gonzalez O. Peña-Axt J. C. Duque-Sanchez E. De Botton-Fernández L. (2018). The language of ethics and double standards in the affective and sexual socialization of youth. Communicative acts in the family environment as protective or risk factors of intimate partner violence. *Front. Soc.* 3:19. 10.3389/fsoc.2018.00019

[B28] Rosell R. L. Martínez I. Flecha A. Álvarez P. (2014). Successful communicative focus groups with teenagers and young people how to identify the mirage of upward mobility. *Qual. Inq.* 20 863–869. 10.1177/1077800414537208

[B29] Searle J. Soler M. (2004). *Lenguaje y Ciencias Sociales. Diálogo entre John Searle y CREA.* Barcelona: Hipatia Press.

[B30] Soler-Gallart M. (2017). *Achieving Social Impact. Sociology in the Public Sphere.* Switzerland: Springer. 10.1007/978-3-319-60270-7

[B31] Vázquez C. Hervás G. Rahona J. J. Gómez D. (2009). Psychological well-being and health. Contributions of positive psychology. *Annu. Clin. Health Psychol.* 5 15–27.

[B32] Wertsch J. V. (1993). *Voices of the Mind. A sociocultural Approach to Mediated Action.* Cambridge, MA: Harvard University Press. 10.2307/j.ctv1pncrnd

[B33] Williams H. Conway M. A. Cohen G. (2008). “Autobiographical memory,” in *Memory in the Real World*, 3rd Edn, eds Cohen G. Conway M. (Hove, UK: Psychology Press), 21–90.

